# Physical Activity Moderates the Relationship Between Screen Time and Body Dissatisfaction in Early Adulthood

**DOI:** 10.1177/10901981251387139

**Published:** 2025-11-06

**Authors:** Rachel Surprenant, David Bezeau, Isabelle Cabot, Jonathan Smith, Hyoun S. Kim, Caroline Fitzpatrick

**Affiliations:** 1 Université de Sherbrooke, Sherbrooke, Québec, Canada; 2 Cégep de Saint-Hyacinthe, Saint-Hyacinthe, Québec, Canada; 3 Cégep Édouard-Montpetit, Longueuil, Québec, Canada; 4 Toronto Metropolitan University, Toronto, Ontario, Canada; 5 University of Johannesburg, Johannesburg, South Africa

**Keywords:** screen time, body dissatisfaction, physical activity guidelines, health-related behavior, young adults, moderation

## Abstract

The transition to adulthood is a vulnerable period for the development of body image issues, which can increase the risk of behavioral disorders such as body dysmorphic and eating disorders. This study explored whether adherence to physical activity guidelines moderates the association between recreational screen time and body dissatisfaction in early adulthood. A sample of 1,475 young adults (mean age 18.81 years, 60.9% female) from 17 French-speaking public colleges in Quebec, Canada, completed self-report questionnaires in Fall 2021 and Winter 2022. Participants reported their daily recreational screen time, engagement in physical activity over the past 3 months, and sociodemographic characteristics. The analysis, based on multivariate linear regression, showed that higher screen time was associated with greater body dissatisfaction, but this relationship was weaker among participants who met the World Health Organization’s physical activity guidelines. These findings suggest that adherence to physical activity guidelines may buffer the negative effects of recreational screen time on body dissatisfaction in young adults, highlighting the value of promoting physical activity in interventions aimed at reducing body dissatisfaction.

Screen time has steadily increased over the last decades, driven by the greater availability and easy access to digital devices and the internet (Wang et al., 2019). In fact, individuals’ daily lives are inextricably linked to screens. Young adults, in particular, spend a considerable amount of time on smartphones and computers. According to recent statistics, 63.8% of Canadian postsecondary students do not respect the recommended limit of 3 hours per day of recreational screen time (Weatherson et al., 2021). Excessive screen time can negatively affect the psychological well-being of young adults, who are already facing new responsibilities, pressures, and challenges (Brown et al., 2022).

The prevalence of body image issues among young adults is also concerning. Approximately 75% of young adults from Australia, Canada, New Zealand, the United Kingdom, or the United States report experiencing body image distress or preoccupations (Milton et al., 2021). This age group exhibits higher levels of body image distress compared with other age groups (Milton et al., 2021). During the transition to emerging adulthood, many experience changes in body appearance and an increase in social comparisons, which can amplify focus on body image and appearance (Grossbard et al., 2009). Furthermore, exposure to high levels of screen time tends to be associated with greater body image concerns (Ryding & Kuss, 2020). Thus, it is essential to identify protective and risk factors for body dissatisfaction among young adults.

Body image is a complex construct that encompasses one’s self-perceptions and self-attitudes related to the body, including thoughts, feelings, behaviors, and beliefs (Cash, 2004). As a component of this multidimensional construct, body dissatisfaction is frequently used as a key evaluative component, reflecting the negative appraisal of one’s body shape, weight, or appearance based on subjective perception (Ferreiro et al., 2014; Lacroix et al., 2023). Body dissatisfaction is also a risk factor for mental health issues such as depressive symptoms, suicidal thoughts, and behavioral disorders, including eating disorders (Crow et al., 2008; Grossbard et al., 2009; Howard et al., 2020). According to the tripartite influence model, media, peers, and parents are primary sources in the development of body dissatisfaction (Hardit & Hannum, 2012). Given that young adults spend a considerable amount of time on recreational screen activities which may include social media (Ryding & Kuss, 2020), their screen use represents an important context for understanding factors related to body dissatisfaction.

Screen time could reduce time spent engaging in activities that support a positive body image, such as physical activity. Research has indicated that physical activity significantly enhances positive body image by improving body-related and overall self-esteem among young adults (Zhuan et al., 2024). In addition, the sense of acquisition of sports body shaping, which refers to feeling stronger or more fit through physical activity, enhances body satisfaction (R. Zhang et al., 2024). Even moderate exercise can contribute to a more positive view of one’s body (Salci & Martin Ginis, 2017). Physical activity also supports social adaptation by increasing social engagement and coping skills, which further promote body satisfaction (Eime et al., 2013). Reducing screen time in favor of physical activity may be a valuable strategy for reducing body dissatisfaction in young adults.

Previous research has primarily focused on body dissatisfaction among young women, who are more likely to engage in risky weight-loss behaviors (Duarte et al., 2015; Howard et al., 2020). However, studies have also indicated that men are concerned about body dissatisfaction, particularly in relation to both weight and muscularity (Bergstrom & Neighbors, 2006; Dal Brun et al., 2024). In addition, body image has been found to be associated with various personal characteristics, including age, disability status, and socioeconomic status (Lacroix et al., 2023; Taleporos & McCabe, 2002; van den Berg et al., 2010; Winter et al., 2021). Much of the recent literature has examined the impact of social media use on body image concerns (Holland & Tiggemann, 2016; Kelly et al., 2018; de Vries et al., 2016). However, other forms of media, such as video games featuring idealized body types, television, and diet and fitness apps, have also been associated with increased body image concerns (Anderberg et al., 2025; Barlett & Harris, 2008; Eisend & Möller-Herm, 2007). These findings highlight the importance of considering overall recreational screen time when examining the relationship between screen use and body dissatisfaction.

A longitudinal study has shown a negative prospective relationship between youth recreational screen time and physical self-concept, which refers to one’s perception of various aspects of their physical self, such as appearance and strength (Babic et al., 2017). In contrast, young adults who engage in physical activity have a more positive body image compared with those who are less active (R. Zhang et al., 2024). Most cross-sectional studies have separately examined the associations between screen use and body dissatisfaction, or between physical activity and body dissatisfaction, which limits our understanding of how these factors interact. Moreover, limited research has simultaneously considered screen time, physical activity, and body dissatisfaction in young adults. Furthermore, physical activity has often been treated as a confounding variable rather than as a potential moderator of the association between screen time and body image (Babic et al., 2017; Hrafnkelsdottir et al., 2022). As such, the present study aims to examine the moderating role of physical activity in the relationship between recreational screen time and body dissatisfaction in young adulthood. In particular, we are interested in whether meeting physical activity guidelines can buffer the negative association between recreational screen time and body dissatisfaction. To achieve this aim, our study draws on a large sample of postsecondary students from Quebec, Canada, a population rarely examined in this literature.

## Method

### Participants

The present study is based on a community-based convenience sample of 1,475 students aged 17 to 24 (mean age = 18.81), recruited in 17 French-speaking colleges in the province of Quebec, Canada. These colleges, who are known as “*collèges d’enseignement général et professionnel*” (CEGEPs), are publicly funded postsecondary educational institutions offering 2-year pre-university programs and 3-year vocational programs. The sample consisted of two cohorts: 815 participants were recruited during the Fall 2021 semester, and 891 participants were recruited during the Winter 2022 semester. Data were collected with self-reported questionnaires administered during the first class of a physical education and health course. The questionnaires covered participant characteristics (e.g., age, sex, disability status, employment status), recreational screen time, physical activity habits, and body dissatisfaction. Most participants reported having no disability or health problems (88.8%) and being employed outside of school (80.3%). The sample included more women (60.9%) than men.

### Procedure

A total of 35 physical education teachers agreed to allow the research team to visit their classes to present the research project to their students and collect data. Depending on the context of the physical education and health course (e.g., outdoor or gymnasium setting), participants completed the questionnaire in French, either on paper or online during class time. All participants provided informed consent and did not receive any compensation for their participation. This study received approval from the ethics review boards of all participating institutions.

### Measures

#### Outcome: Body Dissatisfaction

Participants were asked about their perception of body dissatisfaction using the following items: 1—“My body isn’t pleasant to look at,” 2—“I find myself ugly,” 3—“I don’t like my physical appearance much,” 4—“Nobody finds me attractive,” and 5—“I feel uncomfortable wearing a swimsuit in front of others.” Two items from the original version were reformulated so that all five items reflected greater body dissatisfaction. Responses were reported on a 5-point Likert-type scale ranging from 0 (*strongly disagree*) to 4 (*strongly agree*). A continuous score for overall body dissatisfaction (range 0–20) was computed. Items were derived from the French version called the Physical Self Inventory (Ninot et al., 2000), adapted from the Physical Self-Perception Profile (Fox & Corbin, 1989). In the present sample, internal consistency was high (Cronbach’s α = .89).

#### Predictor: Recreational Screen Time

Participants reported their recreational screen time by answering the following question: “How many hours a day do you usually spend on screens during your free time (outside of school or work)?” (Bado et al., 2022). Participants reported their screen time in hours without predefined response options. To ensure that respondents excluded screen time related to school or work obligations, a separate question was posed about this type of screen time.

#### Moderator: Physical Activity Guidelines Adherence

Participants reported their physical activity during their free time, outside of physical education classes (Doré et al., 2020), during a “typical” week over the 3 months preceding the start of the semester. Physical activity was assessed along two dimensions: duration (minutes per week) and intensity (low, moderate, or vigorous; Warren et al., 2010). Examples were provided to help participants categorize the intensity: low (slight breathlessness, e.g., walking, yoga), moderate (moderate breathlessness, e.g., light jogging, swimming), and vigorous (marked breathlessness, e.g., running, competitive sports; J. Zhang et al., 2021). Following World Health Organization guidelines (WHO, 2020), which recommend a minimum of 150 minutes per week of moderate-intensity endurance physical activity, or a minimum of 75 minutes of vigorous-intensity endurance physical activity, or a combination of both, we calculated total weekly moderate-to-vigorous physical activity (MVPA) for each participant. Participants’ physical activity was then dichotomized to reflect adherence to WHO guidelines (scored as 1) or nonadherence (scored as 0).

#### Covariates

Participants also reported their age in years, sex (coded as 0 = male or 1 = female), disability or health problems status (coded as 0 = no or 1 = yes), and working status (coded as 0 = employed or 1 = unemployed).

#### Data Analysis

First, we conducted a multivariate linear regression analysis to examine the relationship between recreational screen time and body dissatisfaction, adjusting for participant covariates. Then, we examined whether adherence to physical activity guidelines moderated the aforementioned relationship using a simple moderation analysis. A 95% confidence interval (CI) was used to confirm statistical significance. Since screen time and physical activity measurements displayed extreme values, they were therefore winsorized at the 2nd and 98th percentiles. All analyses were conducted with IBM SPSS Statistics 28.

#### Missing Data

Overall, 5.4% of the 1,475 participants had missing data on one of the observed variables. Little’s test was nonsignificant (χ² = 0.971, *df* = 4, *p* = .914) indicating no evidence against data missing completely at random (MCAR) for our predictor (screen time), outcome variable (body dissatisfaction), moderator (physical activity), and covariates (participant age, sex, disability or health problems status, and working status). We used expectation maximization (EM) to handle the missing data. We conducted analyses with multiple imputation and compared them to the analyses conducted on the original sample with missing data. The present analyses are based on complete case selection. As noted by Schafer (1999), a missing rate of 5% or less is generally considered inconsequential.

## Results

### Descriptive Statistics

Table 1 shows sample descriptive statistics for continuous variables and frequencies for categorical variables. Most participants (93%) were aged between 17 and 20 years, and there were more women than men (60.9% vs. 39.1%). On average, participants spent 3.87 hours per day on recreational screen time, and 43.6% of the sample did not meet the physical activity guidelines.

**Table 1.** Descriptive Statistics.

Variable	*M* (*SD*)	Categorical variables (%)	Range
*Participant characteristics*			
Age, *n* = 1,475	18.81 (1.15)		17-24
Sex, *n* = 1,475			
Men		39.1	
Women		60.9	
*Disability or health problems, n = 1,475*			
No		88.8	
Yes		11.2	
*Work status, n = 1,475*			
Employed		80.3	
Not employed		19.7	
*Health-related behaviors*			
Screen time, *n* = 1,470			
Recreational screen time (hours/day)	3.87 (2.23)		0.20-15
*Physical activity guidelines adherence, n = 1,475*			
Not meeting		43.6	
Meeting		56.4	
*Body dissatisfaction*			
Total score, *n* = 1,401	6.70 (5.11)		0-20

### Associations Between Recreational Screen Time, Physical Activity, and Body Dissatisfaction

Associations between recreational screen time, physical activity, and body dissatisfaction are presented in Table 2. Linear regression models adjusted for participant covariates revealed that recreational screen time was positively associated with body dissatisfaction (*b* = .320; 95% CI: .139, .501, *p* < .001). Physical activity guidelines adherence was not directly associated with body dissatisfaction. Age (*b* = .346, 95% CI: .112, .580, *p* < .01) and sex (*b* = .919, 95% CI: .353, 1.485, *p* < .01) were significantly associated with body dissatisfaction, with older participants and young women reporting higher levels of body dissatisfaction.

**Table 2.** Linear Regression Model Estimating Associations Between Recreational Screen Time, Physical Activity, and Body Dissatisfaction (n = 1,396).

	Body dissatisfaction
Variable	*b* (95% CI)
*Health-related behaviors*	
Recreational screen time (hours/day)	.320 (.139, .501)***
Physical activity guidelines adherence (not meeting vs. meeting)	-.190 (-.738, .358)
*Participant covariates*	
Age	.346 (.112, .580)**
Sex (men vs. women)	.919 (.353, 1.485)**
Disability or health problems (no vs. yes)	.419 (-.423, 1.261)
Work status (employed vs. unemployed)	.458 (-.217, 1.133)
*Moderator*	
Recreational Screen Time × Physical Activity Guidelines Status	-.278 (-.520, -.036)*

* denotes *p* < .05. ** denotes *p* < .01. *** denotes *p* < .001.

### Interaction Between Physical Activity, Recreational Screen Time, and Body Dissatisfaction

**Figure 1.**
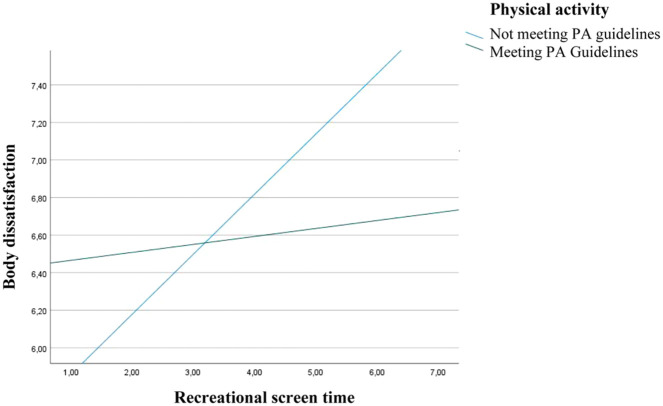
Moderating Effect of Adherence to Physical Activity Guidelines on the Relationship Between Recreational Screen Time and Body Dissatisfaction. *Note.* PA = physical activity.

A significant interaction between recreational screen time and physical activity guidelines adherence was observed (*b* = −.278; 95% CI: −.520, −.036, *p* < .05), indicating that physical activity status moderated the relationship between recreational screen time and body dissatisfaction. Figure 1 illustrates this interaction and shows how the association between recreational screen time and body dissatisfaction differs depending on whether participants meet the physical activity guidelines or not.

## Discussion

With the increasing prevalence of screen time in the daily lives of young adults, it is important to understand the potential psychological impacts of prolonged screen exposure on body dissatisfaction. Our findings indicate that the average recreational screen time of young adults in our sample is 3.87 hours per day, which exceeds the recommended limit of 3 hours per day (Ross et al., 2020). Furthermore, 43.6% of our sample did not meet physical activity guidelines recommended by the WHO (2020). These findings are comparable to previous research conducted with Canadian postsecondary students. In Weatherson et al. (2021), the average daily recreational screen time was 4.7 hours, and 38.9% of participants did not meet physical activity recommendations. Similarly, in Pellerine et al. (2023), the average recreational screen time was 4.75 hours, and 49.6% did not meet physical activity guidelines. Taken together, these findings suggest that our sample exhibits patterns of screen use and physical activity that are broadly consistent with other Canadian postsecondary populations.

Young adults in our sample who were more exposed to recreational screen time were more likely to experience higher levels of body dissatisfaction, and physical activity moderated this relationship. Specifically, the association between screen use and body dissatisfaction was stronger for young adults who did not meet physical activity guidelines. This is consistent with other studies that show higher levels of physical activity have a protective effect on body image dissatisfaction (Monteiro Gaspar et al., 2011). As such, our findings emphasize the importance of considering not only the direct effects of screen time on body dissatisfaction but also the moderating role of physical activity. Given that body dissatisfaction can negatively impact various aspects of a young adult’s life, by diminishing self-worth and increasing the risk of mental health issues and disordered eating behaviors (Howard et al., 2020; van den Berg et al., 2010), our findings underscore the importance of addressing not only screen time but also physical activity to support healthier body image perceptions in young adults. In addition to these main findings, we observed higher body dissatisfaction among women compared with men, which is in line with the literature on sex differences in body image concerns (Quittkat et al., 2019). Consistent with longitudinal research showing that body dissatisfaction increases from adolescence into young adulthood (Bucchianeri et al., 2013), we also found that body dissatisfaction slightly increased with age. These results suggest that both sex- and age-related factors should be considered when designing interventions targeting body image in young adults.

As screen time can reduce the time available for other activities that contribute to the well-being of young adults, future research using longitudinal designs could further explore how the displacement of other essential activities might explain the observed association. For instance, in-person social interactions can improve well-being among young adults (Surprenant et al., 2025; Twenge et al., 2019), which is linked to self-esteem and, in turn, to body dissatisfaction (van den Berg et al., 2010).

The transition to adulthood is an important developmental stage characterized by interpersonal and intrapersonal challenges, such as leaving established social circles, forming new relationships, increasing responsibilities, and achieving financial independence (Eells, 2017; Lanoye et al., 2017). This stage also provides an opportunity to explore new beliefs and behaviors that play a role in personal identity development (Nelson et al., 2008). Given that identity development is a significant process during early adulthood, body dissatisfaction can impact this important transition. Moreover, this period is heavily influenced by the social environment, with parents taking on a less prominent role. In this context, exposure to screens can affect both identity development and body dissatisfaction. Therefore, future research should consider specific content of screen use, such as social media versus other online activities, to better identify which are most closely linked to body dissatisfaction in young adults.

The cross-sectional design of our study is a limitation, as it prevents us from determining the direction of association. For instance, it may be the case that young adults with greater body dissatisfaction choose to devote more of their time to recreational screen use. In addition, a bidirectional relationship may exist between social media engagement and body image outcomes (Cohen et al., 2017). Indeed, the U.S. Surgeon General has recently emphasized the importance of regularly considering the potential for bidirectional relationships when investigating the links between screen media use and mental health in youth (U.S. Department of Health and Human Services, 2023). Longitudinal studies with repeated measures of screen use and youth body image reports would be ideally suited to clarify the direction of this association. We relied on self-reported measures of recreational screen time, physical activity, and body dissatisfaction, which may have led to social desirability bias or shared measurement error. Specifically, reports of MVPA may have been overestimated by participants (James et al., 2016). As such, future studies using accelerometers are warranted. In addition, our measure of screen time did not account for the content or context of screen exposure, which could influence the associations observed in this study. For example, heavy social media use has been shown to exacerbate body dissatisfaction by exposing young adults to ideal body internalization and unfavorable social comparisons (Thai et al., 2024). Finally, the use of a convenience sample limits the generalizability of our findings. Future research should aim to replicate these results with more diverse and representative populations.

The main strength of this study is its ability to simultaneously examine screen time, physical activity, and body dissatisfaction among young adults in a large sample. To date, most research has focused either on screen use or physical activity in relation to body dissatisfaction. Another strength of our study is that we were able to identify potentially modifiable determinants of body dissatisfaction in young adulthood, such as physical activity.

Implications and recommendations can be drawn from our findings to address the negative effects of excessive recreational screen time on body dissatisfaction among young adults. For example, promoting the reduction of screen time and encouraging regular physical activity could be key. Practical interventions should aim to help young adults integrate physical activity into their daily routines and promote accessible opportunities provided by postsecondary institutions, such as fitness classes, intramural sports, and outdoor programs. These activities can enhance physical competence, self-esteem, mood, and social support, which may buffer the association between high screen time and body dissatisfaction (Eime et al., 2013; R. Zhang et al., 2024). Health practitioners and professionals can also collaborate with young adults to both encourage participation in these physical activity programs and limit screen time. In support of this approach, an experimental study, provides evidence that encouraging university students to limit social media use to 30 minutes per day can improve well-being (Hunt et al., 2018). Early interventions targeting screen time habits in childhood may also have lasting effects into young adulthood (Wahi et al., 2011). Addressing screen time from a young age may therefore lead to sustained benefits as children grow, potentially helping to prevent body dissatisfaction in early adulthood. Finally, public health campaigns should encourage the avoidance or limitation of screen time in favor of activities that contribute to the well-being of young adults.

Given that a large proportion of young adults report experiencing body image distress or preoccupations, body dissatisfaction remains a widespread and concerning issue during the transition to adulthood (Milton et al., 2021). It increases further during the transition to young adulthood (Bucchianeri et al., 2013). Body dissatisfaction can negatively impact young adults’ self-esteem and mental well-being during the development of identity in this stage of life. As screen use continues to increase among young adults, it is essential to explore its effects on well-being and body image. Our results suggest that excessive screen time is linked to higher body dissatisfaction, while physical activity serves as a protective factor, moderating this relationship. These findings highlight the importance of integrating physical activity promotion into interventions aimed at improving well-being in this population.
